# Using Next Generation Sequencing for Multiplexed Trait-Linked Markers in Wheat

**DOI:** 10.1371/journal.pone.0143890

**Published:** 2015-12-01

**Authors:** Amy Bernardo, Shan Wang, Paul St. Amand, Guihua Bai

**Affiliations:** 1 Department of Plant Pathology, Kansas State University, Manhattan, Kansas, United States of America; 2 Department of Agronomy, Kansas State University, Manhattan, Kansas, United States of America; 3 United States Department of Agriculture, Agricultural Research Service, Hard Winter Wheat Genetics Research Unit, Manhattan, Kansas, United States of America; USDA-ARS-SRRC, UNITED STATES

## Abstract

With the advent of next generation sequencing (NGS) technologies, single nucleotide polymorphisms (SNPs) have become the major type of marker for genotyping in many crops. However, the availability of SNP markers for important traits of bread wheat **(** *Triticum aestivum* L.) that can be effectively used in marker-assisted selection (MAS) is still limited and SNP assays for MAS are usually uniplex. A shift from uniplex to multiplex assays will allow the simultaneous analysis of multiple markers and increase MAS efficiency. We designed 33 locus-specific markers from SNP or indel-based marker sequences that linked to 20 different quantitative trait loci (QTL) or genes of agronomic importance in wheat and analyzed the amplicon sequences using an Ion Torrent Proton Sequencer and a custom allele detection pipeline to determine the genotypes of 24 selected germplasm accessions. Among the 33 markers, 27 were successfully multiplexed and 23 had 100% SNP call rates. Results from analysis of "kompetitive allele-specific PCR" (KASP) and sequence tagged site (STS) markers developed from the same loci fully verified the genotype calls of 23 markers. The NGS-based multiplexed assay developed in this study is suitable for rapid and high-throughput screening of SNPs and some indel-based markers in wheat.

## Introduction

Single nucleotide polymorphisms (SNPs) are the most abundant form of genetic variation and provide a rich source of DNA markers [1]. SNPs are generally bi-allelic in nature, and have a low mutation rate [2,3], making them an ideal marker of choice for genetic and physical mapping, genome-wide association studies, phylogenetic analysis, genomic studies, and marker-assisted selection (MAS) [4,5,6,7]. A SNP in a genic or promoter region may be responsible for a change in phenotype and can serve as a functional marker for a trait of interest in MAS [8,9].

With the advent of next generation sequencing (NGS) technologies, SNPs have become the most frequently used type of marker for genotyping in many crops such as rice, corn, barley and sorghum [10,11,12,13]. In wheat, SNP development is hampered by the large genome size, highly repetitive elements (>80%), and by three different, but closely related, genomes among which corresponding genes share a high level of sequence similarity. Therefore the majority of the markers used for wheat genotyping have been simple sequence repeats (SSR) and sequence tagged site (STS) markers [14,15]. A shift in the use of SNP markers in wheat breeding has been slow even though a large number of wheat SNPs have been developed [6,16,17,18].

For wheat MAS, "kompetitive allele specific PCR" (KASP) genotyping assays have been developed for resistance genes to leaf rust (*Lr21*), soil-borne mosaic virus (SBMV), and preharvest sprouting [19,20,21,22]. The KASP assay is a uniplex, fluorescence-based genotyping technology based on allele-specific oligo extension and fluorescence resonance energy transfer for signal generation [23]. KASP assays can detect SNPs, some indels, and cleaved amplified polymorphic sequence (CAPS) sites. Target alleles are detected based on the fluorescence of the dye-linked amplicons. The TaqMan assay is also usually uniplex and uses two PCR primers and two allele-specific probes and can detect SNPs, some indels, and CAPS sites [24]. Attached to the probe is a fluorescent dye and a quencher that suppresses dye fluorescence. When a probe binds to a target site between two primers, the 5’ to 3’ exonuclease activity of the *Taq* polymerase enzyme cleaves the probe and leads to the separation of the dye from the quencher resulting in fluorescence. TaqMan assays for leaf rust resistance (*Lr34* and *Lr37*) were reported for wheat MAS [14,25,26]. Another genotyping platform is SNaPshot, a single-base extension assay based on the fluorescence of a dye-linked extended nucleotide. This genotyping method can be multiplexed if primers are designed with increasing lengths, but usually only up to a maximum of 10-plex. In wheat, only uniplex SNaPshot assays of *Fhb1* and *TaPHS1* have been reported [20,27]. The drawback of TaqMan, KASP and SNaPshot is that they are usually uniplex assays or have a very low level of multiplexing. Fluidigm’s Dynamic Array Integrated Fluidic Circuit (IFC) is a chip-based genotyping system that performs TaqMan, KASP or Fluidigm SNP Type assays in nanoliter volumes to reduce genotyping costs [28].

As more markers become available for routine MAS, a shift from uniplex to multiplex assays is essential. In addition, breeders are more interested in moving from conventional to molecular breeding because phenotyping is relatively labor-intensive, time-consuming, and expensive. In contrast, quickly evolving NGS-based genotyping technologies facilitate rapid genotyping throughput and decreasing cost per data point. Moreover, MAS is usually done during the early stages of plant growth cycles and only those plants with positive markers are kept for further phenotyping which saves resources. Multiplex marker assays allow the simultaneous analysis of multiple markers for different traits which increases MAS efficiency [29].

Several multiplex platforms are available for marker assays. The Sequenom MassArray is a genotyping platform that can multiplex up to 40 markers per sample run [30]. It involves the multiplex amplification of target primers by single-base extension, followed by allele detection based on the molecular weight of the extension products [30]. Adjustment of the extension primer concentration is essential to generate products of uniform peak heights [31] and the MassArray extension products need to have differing masses within the 4,500 to 9,000 Da detection window to differentiate the peaks [30]. Masouleh et al. [31] used MassArray to assay gene-linked SNPs in rice. In wheat, Berard et al. [32] used MassArray to validate the genotyping calls from 11 SNPlex markers and observed a 96% agreement.

Another multiplex platform is the high-density SNP array. In wheat, the newly developed 9K and 92K wheat SNP chips contain well-distributed gene-based SNPs and have been widely used for germplasm characterization and quantitative trait locus (QTL) mapping [17,18]. Although Sequenom arrays and SNP chips provide ideal throughput, assays have a high per-sample cost [33]. Genotyping-by-sequencing (GBS), which involves sequencing of DNA fragments representing partial, but genome-wide coverage of samples, can solve these problems. In GBS, DNA complexity is reduced by restriction digestion of DNA to generate fragments which are then ligated to adapters for sequencing. The resulting sequence data can be used for both SNP discovery and genotyping [6,34]. A drawback of GBS genotyping is that it has a high percentage of missing data due to low sequence coverage. These high-throughput systems are all good platforms for SNP discovery, genomic selection, and genome-wide association studies; however, they may not be a cost-effective genotyping platform for MAS which needs a quick turnaround time, low per-sample cost, and a very low rate of missing data. MAS usually uses a set of specific markers for specific QTLs and genes at a medium throughput rather than random genes or markers.

More recently, a multiplexed genotyping platform that combines multiplexed PCR and multiplexed samples using barcodes with NGS has been described as genotyping by multiplexing amplicon sequencing (GBMAS) [35]. A similar procedure was referred to as spiked GBS (sGBS) (Rife et al., 2015) in which they sequenced a multiplex PCR of a specific marker set together with a GBS library. sGBS allows the multiplex genotyping of markers linked to important QTL at a fraction of the cost of an NGS run, thus it is economical [36]. As long as the two combined libraries use different barcodes, sequences from the libraries can be separated during data analysis. Sequencing entire SNP-harboring fragments also allows the genotyping of additional SNPs, indels, and unique multiple base changes in the fragments, if present, which is not possible in other SNP genotyping systems. Moreover, the identification of novel genetic changes is not a hindrance, nor does it need additional allele-specific primers.

In wheat, dozens of SNPs that are tightly linked to important genes or QTL have been reported to date. There are also CAPS and indel-based STS markers linked to, or within, wheat genes of breeders’ interest. Multiplex assays for these marker sites, however, are still not available. A rapid increase in the number of diagnostic or tightly linked SNPs is expected in the near future because extensive efforts have been made in QTL mapping using SNP markers. Thus multiplex assays of these SNPs will facilitate effective use of existing and new SNPs in MAS. This study aimed to develop such an assay for a set of SNPs and some indel-based markers linked to QTLs and genes for important traits in hard winter wheat (HWW) using NGS and evaluate the robustness of GBMAS in wheat MAS.

## Materials and Methods

### DNA Samples

We used 24 wheat genotypes each carrying at least one of the following genes: plant height (*Rht-B1b)*, disease or insect resistance including resistance to Fusarium head blight (*Fhb1*), leaf rust (*Lr21*, *Lr42*, or slow rusting), stem rust (*Sr35*, *Sr39*, or *Sr40*), adult plant leaf diseases (*Lr34/Yr18/Pm38/Sr57*, *Lr46/Yr29/Pm39/Sr58*, *Sr2/Yr30*, or *Yr17/Lr37/Sr38*), wheat streak mosaic (*Wsm2*), wheat soil-borne mosaic virus, Hessian fly (*HF1A* or *HF6B*), quality traits such as high grain protein content (*HGPC/Yr36*), pre-harvest sprouting (*TaPHS1* or *PHS4A*) or gluten strength (*GluB1* or *GluD1*).

Seeds were planted in a plastic growing tray containing Metro-Mix 360 growing medium (Hummert Int., Earth City, MO) and seedlings were grown in a greenhouse at 15°C with a 12 h light/dark cycle. Genomic DNA was extracted from very young leaf tissues of single plants (four replications/genotype) at the 3-leaf stage using an SDS method (Pallotta et al, 2003). The extracted DNA was not treated with RNAse. DNA was quantified using a Quant-iT^™^ PicoGreen^®^ dsDNA assay kit (LifeTechnologies, Carlsbad, CA).

### Primer Design

**Fig 1:**
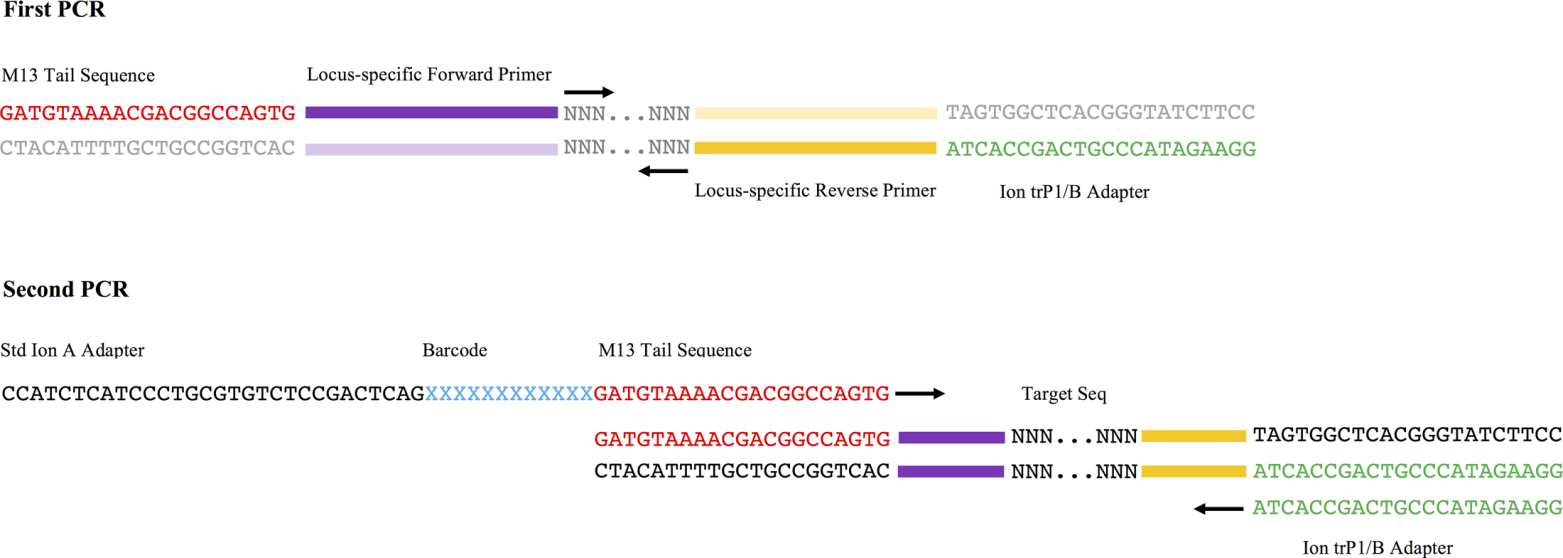
GBMAS two-step PCR. The first PCR is a multiplex and amplifies the different target regions containing SNP(s). The locus-specific forward primers were tailed with an M13 sequence at the 5’-end and the reverse primers were tailed with Ion truncated P1/B sequence. The generated amplicons contains an M13 tail and Ion truncated P1/B sequence. The second PCR involves the addition of barcodes and standard Ion A adapter to the amplicons.

Thirty-three locus-specific primer pairs of 17–21 nucleotides each were designed using the Sequenom MassArray Assay Design 4.0 software (Sequenom, San Diego, CA) with the amplicon length set at 150 nucleotides as the optimum, ranging from 80 to 200 nucleotides (S1 Table). The primers were modified (Fig 1) by adding barcodes and specific sequences compatible with the Ion Torrent Proton System (LifeTechnologies, Carlsbad, CA). The locus-specific forward primers for the first PCR were tailed with an M13 derived sequence (GATGTAAAACGACGGCCAGTG) at the 5’-end to enable the addition of barcoded adapters during the second round of PCR. The Ion truncated P1/B adapter sequence (CCTCTCTATGGGCAGTCGGTGAT) was concatenated to the 5’-end of the locus-specific reverse primers. For the second PCR, the forward fusion primer consisted of, from 5’ to 3’, the standard Ion A adapter sequence (CCATCTCATCCCTGCGTGTCTCCGACTCAG), a unique barcode with 10–12 nucleotides, followed by the M13 tail sequence. A combination of different barcodes with the M13 tail gave us the flexibility to multiplex the same set of markers in different samples. The reverse primer for the second PCR was the Ion truncated P1/B adapter sequence. Ninety-six unique barcodes from LifeTechnologies were used to tag the 24 wheat accessions under four different PCR conditions.

### Library Preparation

Library preparation was done following the protocol from the Schnable Lab with some modifications [35]. The reaction mix for the first PCR of 10 μl total volume consisted of 1X LGC PCR buffer containing 1.8 mM MgCl_2_ (LGC Genomics, Beverly, MA), 1.325 mM additional MgCl_2_, 500 μM each dNTP, a primer mix (described below), 1 unit KlearTaq (LGC Genomics, Beverly, MA) and template DNA. The primer mix consisted of 33 sets of locus-specific primers. Genomic DNA concentrations ranging from 17 to 141 ng were tested. PCR using different primer concentrations (from 2 to 100 nM) and PCR protocols [touchdown (TD) and non-TD] were evaluated.

The TD program started at 94°C for 10 min, followed by 10 cycles of 20 s at 94°C, 1 min at 64°C (-0.8°C per cycle in each subsequent cycles), 30 s at 68°C, and 20 cycles of 20 s at 94°C, 1 min at 56°C, 30 s at 68°C, and a final extension of 3 min at 72°C. The non-TD program consisted of 10 min at 94°C, 50 cycles of 20 s at 94°C, 1 min at 56°C, 30 s at 68°C, and a final elongation time of 3 min at 72°C. The first PCR product from each wheat sample was diluted with 10 μl ddH_2_O and 2 μl of the diluted product was used as the template for the second PCR. The 5 μl second PCR mix contained 1X LGC PCR buffer with 1.8 mM MgCl_2_ (LGC Genomics, Beverly, MA), 1.2 mM additional MgCl_2_, 500 μM dNTPs, 400 nM forward fusion primer containing a barcode and 400 nM Ion truncated P1/B reverse primer, and 1 unit KlearTaq (LGC Genomics, Beverly, MA), and ran using the following PCR profile: 10 min at 94°C, 15 cycles of 15 s at 94°C, 30 s at 60°C, 1 min at 72°C, plus a final extension step of 3 min at 72°C. Four pools (libraries) of 24 samples (4 μl PCR product per sample) were made using either high or low primer concentrations and using either TD or non-TD PCR programs. All of the libraries were quantified using the Qubit^®^ dsDNA HS assay kit (LifeTechnologies, Carlsbad, CA).

### Product Purification and Sequencing

The libraries were filtered using a Nanosep 10K Omega Ultrafiltration membrane (Pall Corporation, Port Washington, NY) and then run on a 2% E-Gel^®^ SizeSelect^™^ Gel (LifeTechnologies, Carlsbad, CA) to select PCR fragments from 150 to 300 bp. Purified libraries were quantified using the Qubit^®^ dsDNA HS assay kit (LifeTechnologies, Carlsbad, CA), diluted to the appropriate concentration as recommended by LifeTechnologies and equimolar pools of the libraries were spiked into a GBS library at 3% by concentration. Sequencing was done using an Ion Torrent Proton Sequencer with PI v2 chips (LifeTechnologies, Carlsbad, CA).

### Data Analysis

The analysis pipeline for GBMAS included four steps. First, sequence reads were sorted according to barcodes to separate different wheat accessions and then the barcodes were removed. Second, the sequence of the forward primers were used to further subdivide the reads within each wheat accession into different markers. These steps were performed using FLEXBAR version 2.5 [37]. Third, sequences were aligned to the reference sequences using BLAT, a BLAST-like alignment tool [38], at the default alignment settings. Each marker reference sequence consisted of the DNA sequence for the amplicon from the known positive wheat accession, and the base position for each base of interest was annotated as part of the marker name. Fourth, based on the sequence alignment results of each wheat accession-marker combination, the total number of aligned reads containing A, C, G, and T bases, and N (null allele or a deletion) in the base position of interest, was summarized using a custom Perl script. The percentage of favorable allele (the allele of interest, e.g. an allele that is associated with disease resistance) reads was then calculated by dividing the favorable allele count by the total number of sequence reads and then multiplying by 100. The percent favorable allele reads of the positive control DNA for a particular marker was used as the cutoff value for homozygous positive. Any sample with a percent favorable allele read equal to or greater than the positive control was considered homozygous positive for the marker and samples with a percentage less than the cutoff value were genotyped as homozygous negative.

### Marker Validation

Markers designed for GBMAS were validated using KASP and STS markers. For the STS markers, each 10 μL PCR reaction contained: 1X ammonium sulfate buffer (Bioline, Randolph, MA), 2.5 mM of MgCl_2_, 200 mM of each dNTP, 50 nM of forward M13-tailed primer, 100 nM of reverse primer, 30 nM of dye-labeled-M13 (FAM, VIC, HEX or NED) fluorescent primer, 60 ng DNA, and 1 unit *Taq* polymerase (Promega, Madison, WI). PCR was performed using a PTC-200 thermal cycler (Bio-Rad Labs, Hercules, CA) using a touchdown program starting at 96°C for 5 min; followed by 5 cycles of 1 min at 96°C, 3 min at 68°C (-2°C per cycle in subsequent cycles), and 1 min at 72°C; 5 cycles of 1 min at 96°C, 2 min at 58°C (-2°C per cycle in subsequent cycles), and 1 min at 72°C; 40 cycles of 20 s at 96°C, 20 s at 50°C, and 30 s at 72°C; and a final extension of 5 min at 72°C.

All STS PCR products were mixed with Hi-Di formamide and GeneScan 500 size standard (Life Technologies, Carlsbad, CA). Fluorescence-dye-labeled PCR products were visualized on an ABI3730 sequencer (Life Technologies, Carlsbad, CA) and were scored using GeneMarker V1.5 (SoftGenetics, State College, PA). The *csSr2*-linked marker is a CAPS marker, and its amplicons were digested with *Bsp*HI enzyme (New England BioLabs, Ipswich, MA) before they were analyzed in the ABI3730 sequencer. The digestion reaction mix containing 1X CutSmart buffer, 10 μl PCR product and 2.5 U *Bsp*HI was incubated at 37°C for 2 h then at 80°C for 20 min to deactivate the enzyme.

KASP reactions were done following the manufacturer’s protocol (LGC Genomics, Beverly, MA) using 50 ng DNA in a 5 μl reaction volume. Prior to PCR, plates were scanned for background fluorescence using an ABI7900HT Fast Real-Time PCR System (Life Technologies, Carlsbad, CA). The reactions were run on an iCycler (BioRad, Hercules, CA) with the following PCR program: initial denaturation at 94°C for 15 min followed by 10 cycles of 94°C for 20 s, 1 min at 65°C (-0.8°C per cycle in subsequent cycles), followed by 40 cycles of 20 s at 94°C and 1 min at 57°C. After PCR, the plates were read in an Applied Biosystems 7900HT System and data were analyzed using the "Allele Discrimination" method of the Sequence Detection System software v2.4 (Life Technologies, Carlsbad, CA).

## Results

### Effect of DNA Concentrations on Sequence Read Counts

**Fig 2:**
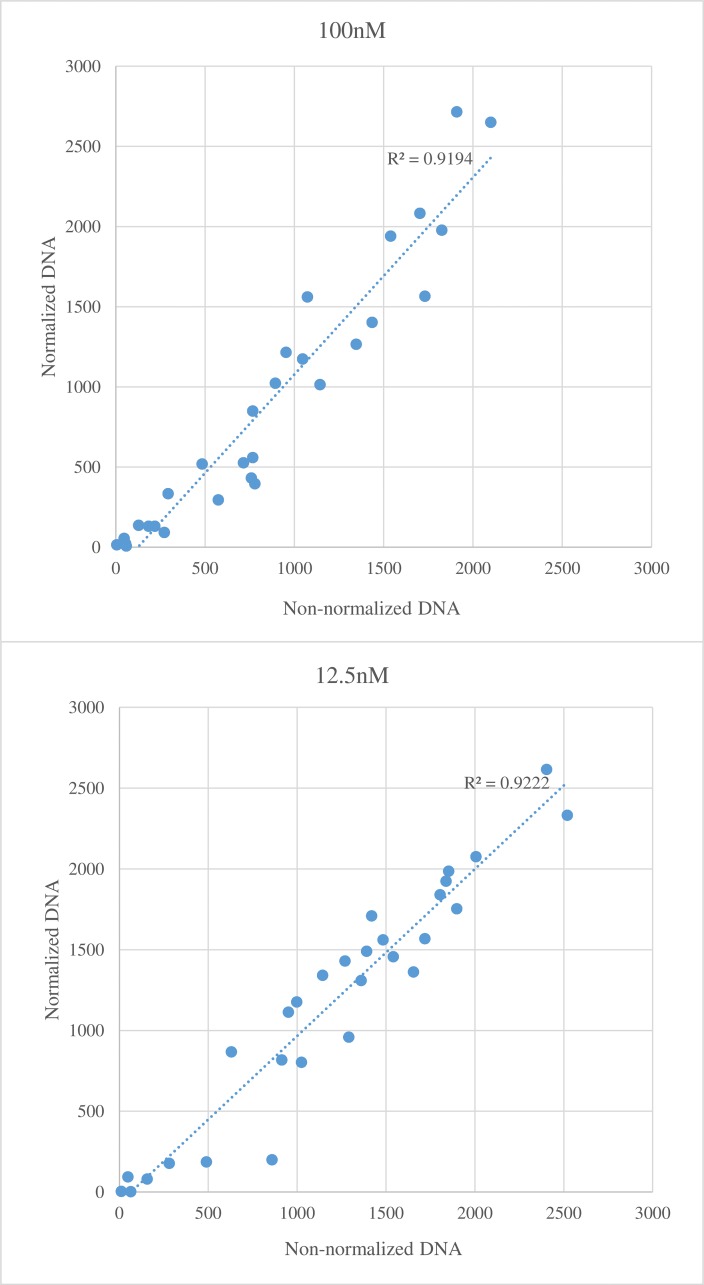
Average number of reads per marker analyzed using non-normalized (x-axis) and normalized (y-axis) DNA. The top figure used 100 nM primers for PCR and the bottom figure used 12.5 nM primers for PCR.

DNA concentrations typically vary among samples even when samples are processed from the same amount of tissue at the same time. To determine if normalization of template DNA concentration is essential for GBMAS, the average number of reads per marker were compared between non-normalized and normalized DNA at two primer concentrations (12.5 nM and 100 nM, Fig 2). The DNA concentrations for non-normalized template DNA ranged from 17 to 141 ng with a mean of 68 ng per reaction. The normalized samples contained 60 ng DNA per PCR reaction. The correlation in read counts between normalized and non-normalized DNA was very high (R^2^ ~0.92) at both primer concentrations (Fig 2), suggesting that normalization is not necessary at the concentration range used in this study.

**Fig 3:**
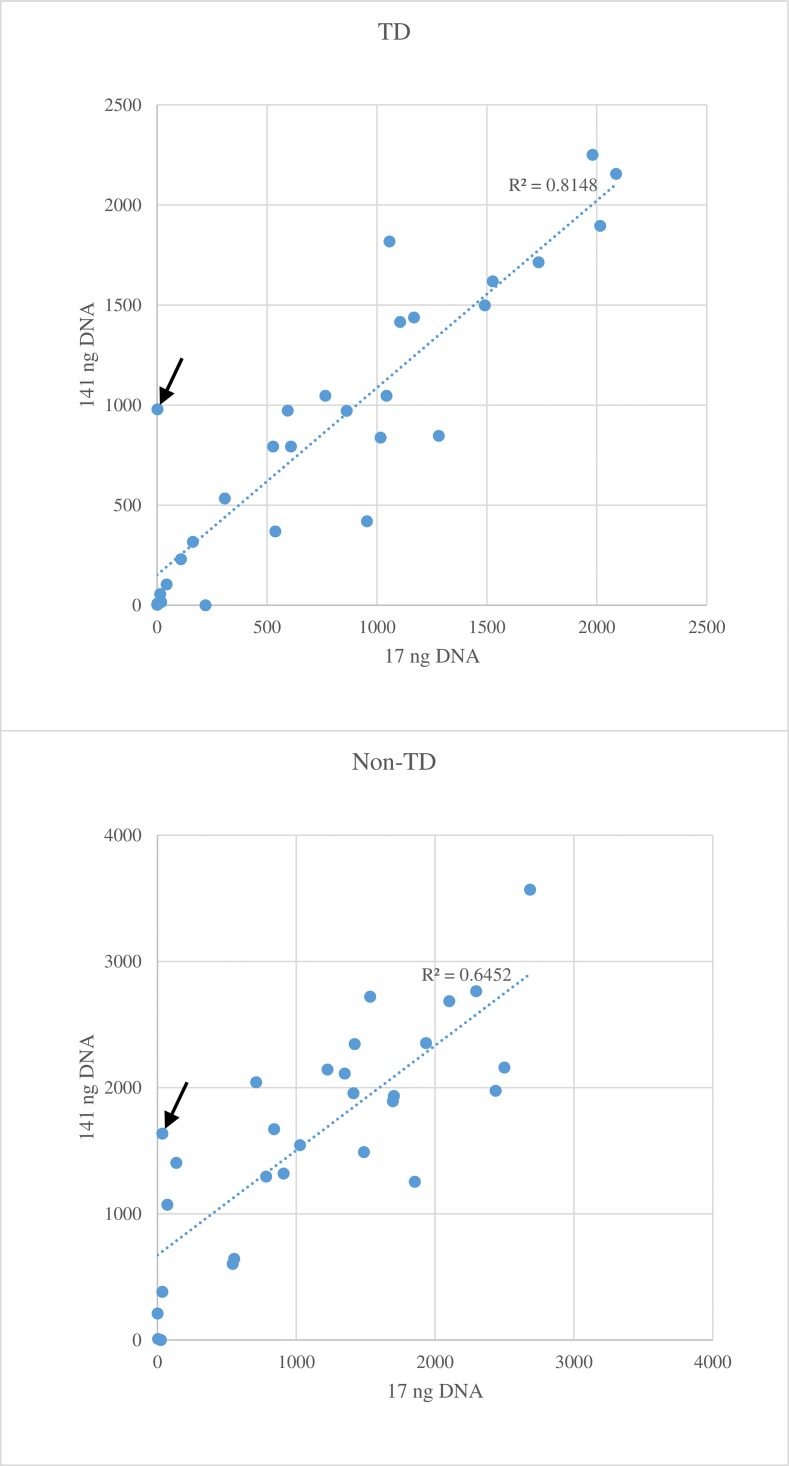
Average number of sequence reads per marker using 17 ng (x-axis) and 141 ng DNA (y-axis) as template. The top figure used touch down (TD) and the bottom figure used non-TD PCR. The arrow points to the number of reads of CNL9 marker for *Sr35*.

A higher correlation of sequence read numbers between high (141 ng) and low (17 ng) DNA concentrations was observed for TD PCR (R^2^ = 0.81) than non-TD (R^2^ = 0.65, Fig 3). On average, samples with a higher DNA concentration yielded more reads than those with a lower concentration, thus TD PCR was slightly more tolerant to low DNA concentrations. The biggest discrepancy in the number of reads occurred using marker CNL9 for *Sr35* because the marker was detected only in the sample with a high DNA concentration and not in the low concentration sample (null allele) (Fig 3). For most markers in this study, the number of reads generated from the 17 ng DNA template was sufficient. Three markers (Lr21_indel_R, PHS4A_3743_9, and Umn10) with very low read counts were not successfully converted to GBMAS.

### Effect of Primer Concentrations on Read Counts

**Fig 4:**
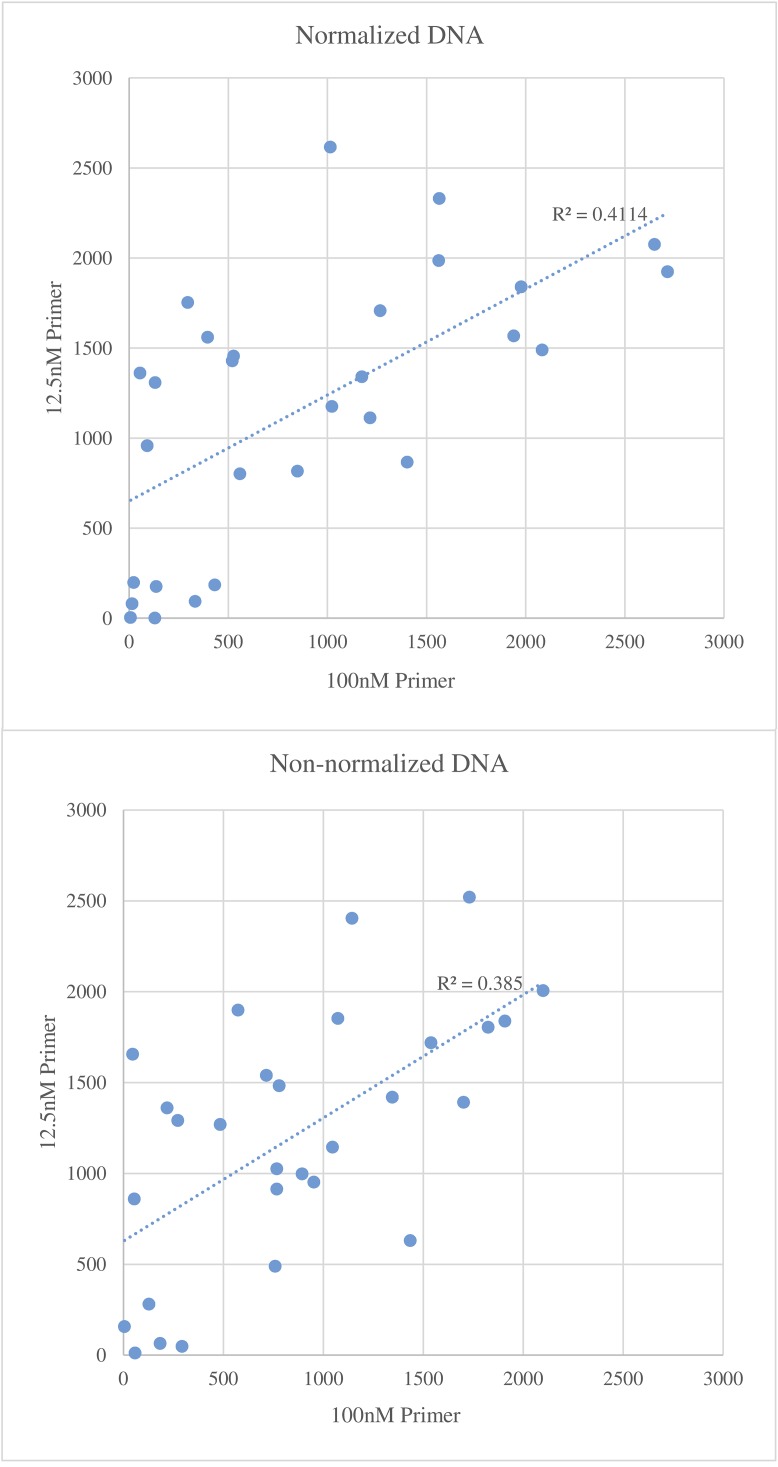
Average number of reads per marker as affected by low (y-axis) or high primer concentrations (x-axis). The top figure used normalized and the bottom figure used non-normalized DNA.

The effect of primer concentrations (12.5 nM and 100 nM) on the mean number of read counts is shown in Fig 4. In general, the lower primer concentration gave higher read counts for more markers than the higher primer concentration regardless of whether DNA concentration was normalized or not. The relatively low R^2^ values (< 0.42) indicate that primer concentration has a large effect on the number of reads and warrants optimization. A combination of different primer concentrations may help maximize read counts.

**Fig 5:**
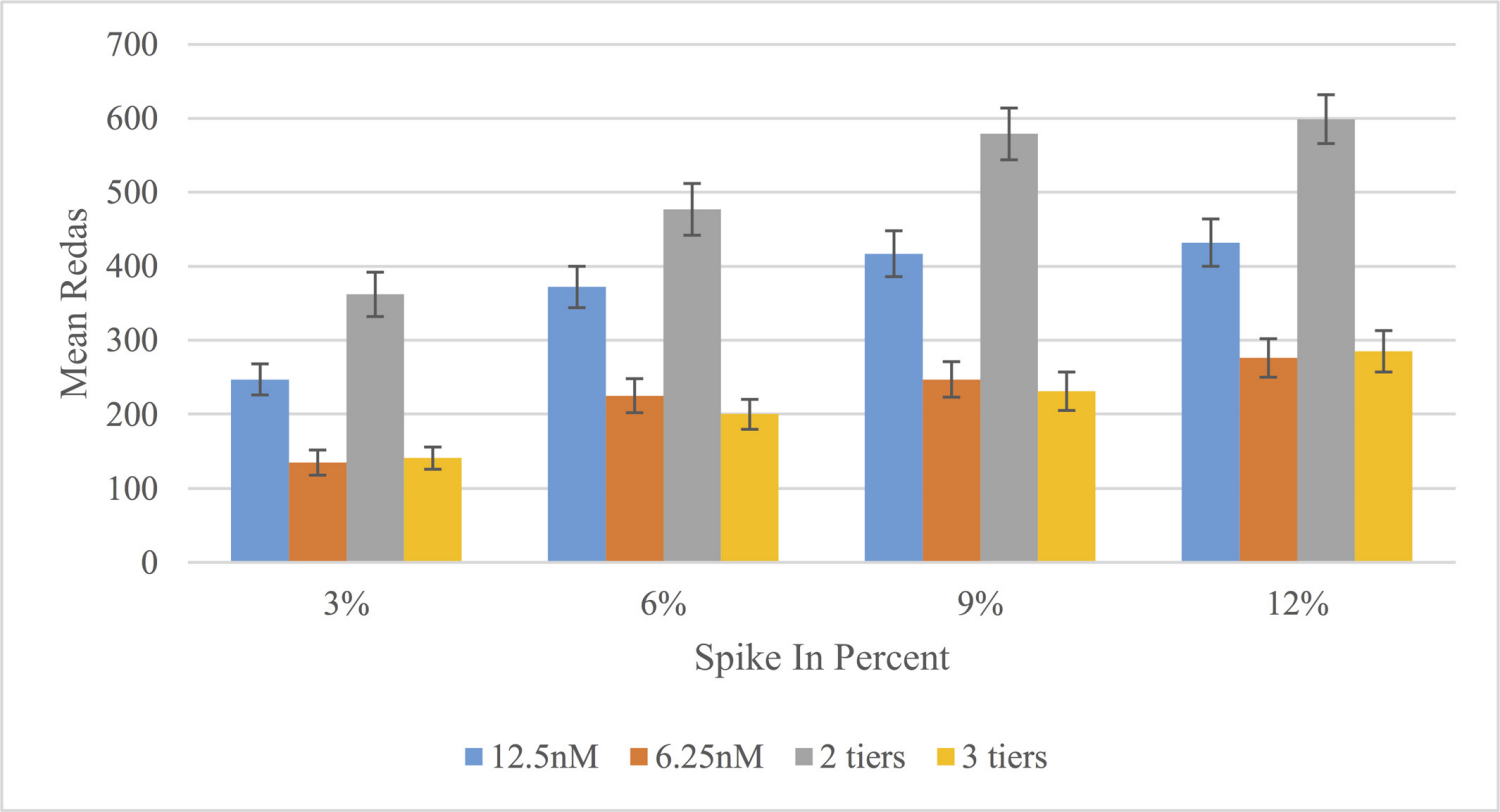
Average number of reads as affected by different primer concentrations: 6.25 nM, 12.5 nM, 2 tiers of 2 nM and 25 nM, and 3 tiers of 2 nM, 15 nM and 30 nM. Standard errors are shown as error bars on top of columns.

To determine an optimum primer concentration for more uniform GBMAS read counts, four combinations were evaluated: two relatively low concentrations (6.25 nM and 12.5 nM), and combinations of two (2 tiers of 2 nM and 25 nM) or three (3 tiers of 2 nM, 15 nM, and 30 nM) different concentrations for different markers based on initial test results. Average read counts were highest for 2 tiers, followed by the 12.5 nM primer set, across all of the spike-in percentages tested (Fig 5). The other two primer combinations had read counts less than half of that for the 2 tiers. Thus the 2 tier primer set was further optimized and adjusted to an 8 nM and 16 nM pool.

### Effect of PCR Profiles on Read Counts

**Fig 6:**
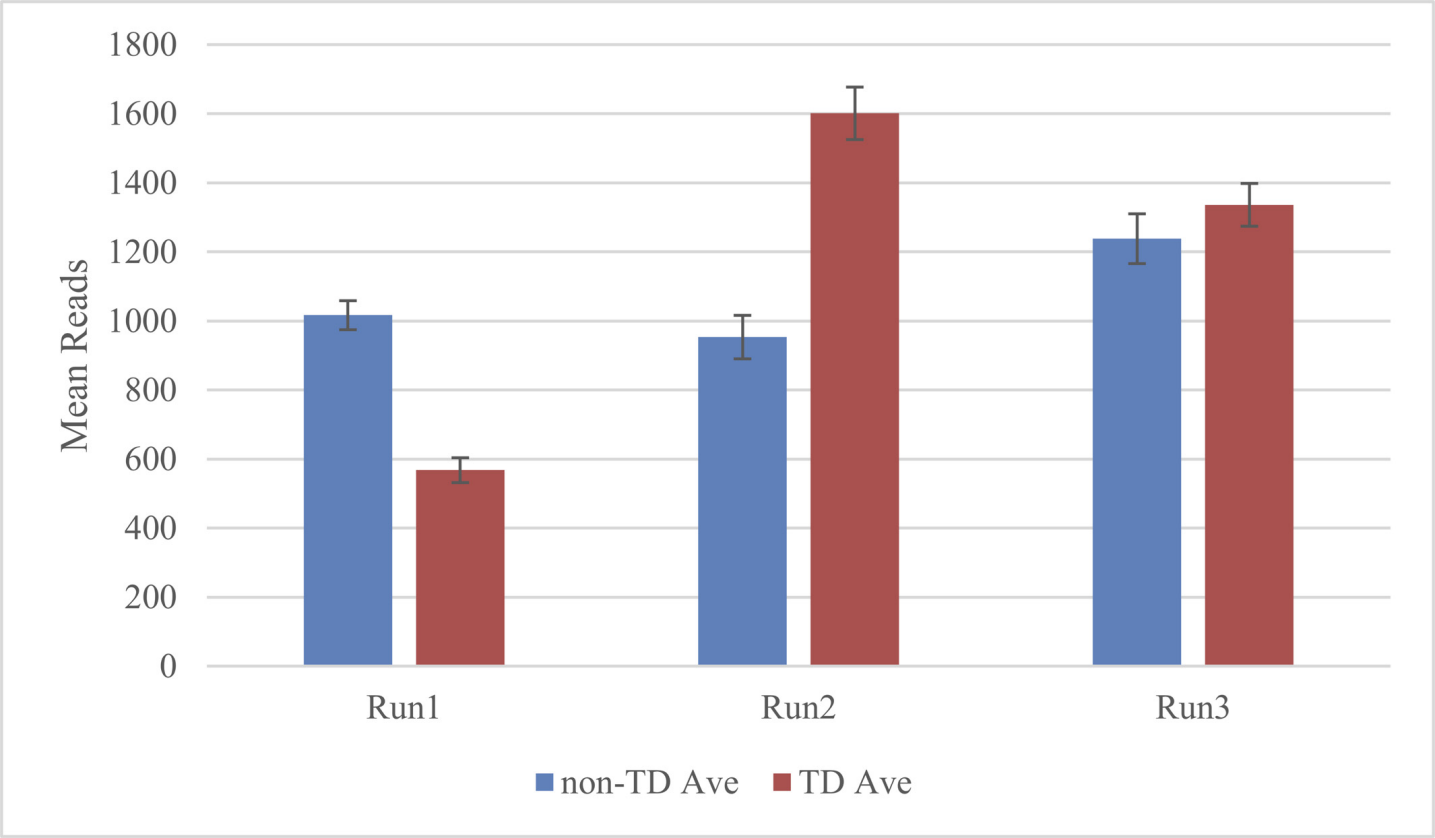
Average number of reads per marker in non-TD (blue column) and TD PCR (red column). Standard errors are shown as error bars on top of columns. Samples were run using the primer combination of 8 nM and 16 nM.

Fig 6 shows the average sequence read counts per marker after read quality filtration. The non-TD profile gave a more consistent number of read counts across runs than the TD profile. Also, the non-TD profile increased the uniformity of minimum read counts for each sample, whereas the TD profile decreased the uniformity of minimum read counts across tested samples (data not shown).

The average sequence read count per marker for the best treatment combination of 2 tier primer pool with non-TD PCR was 889. The number of reads varied greatly by marker, ranging from 0 to 3565; 10 reads per allele were used as a minimum read cutoff to call allele genotypes. Three markers were dominant. The marker Bx7oe was developed based on an indel mutation, and the wild type allele is a null-allele, thus wild type wheat samples did not generate any read. Likewise, the two alien fragment-derived stem rust markers for *Sr2* and *Sr35* also generated zero reads in wheat samples that do not possess the alien fragments.

### Minimum Cutoffs for Favorable Alleles

**Fig 7:**
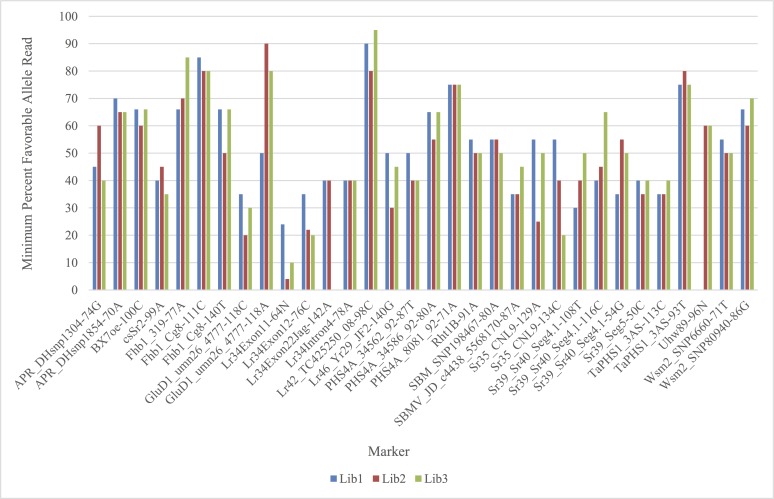
Minimum percentage of favorable allele reads from three GBMAS libraries constructed using the 2-tier primer pool of 8 nM and 16 nM primers and non-TD PCR.

Fig 7 shows the minimum percentage of favorable allele reads in three GBMAS libraries constructed using the two-tier primer pool (8 nM and 16 nM) and non-TD PCR. Because the samples used in this study are all homozygous wheat varieties, the minimum percentage of favorable allele reads of the positive control can be used as the read percentage cutoff for classifying homozygous positive genotypes. Usually, these percentage values varied with markers and also across runs with two exceptions, Lr34Inron4 and PHS4A_8081_92 gave consistent cutoff values of 40% and 75%, respectively, in all libraries. For other markers like Sr35_CNL9 and Sr39_Sr40_Seg4.1, the minimum percentage of reads for favorable alleles varied from 50% to 33%, which matches with the theoretical percentage of an expected allele amplified from two or three genomes (Table 1).

**Table 1:** Primer specificity and theoretical read percentages of expected favorable SNP allele (A) amplified from one to three genomes of wheat.

Primer Specificity	Genome 1	Genome 2	Genome 3	% Favorable Allele (A) Reads
**Genome-specific**	AA			100
	AT			50
	TT			0
**Semi-genome-specific**	AA	TT		50
	AT	TT		25
	TT	TT		0
**Non-genome-specific**	AA	TT	TT	33
	AT	TT	TT	16
	TT	TT	TT	0

The wild type SNP allele in this example is T.

### Marker Conversion and Call Rates

Among 33 markers tested, 27 were successfully converted into GBMAS markers (82%) and six (HF1A-5150, Lr21_indel_R, Lr42-113325_01, PHS4A_3743_9, Umn10 and Yr17_Lr37_Sr38) did not consistently produce enough sequence reads. Based on the percentage of samples for which genotypes can be confidently scored in GBMAS analysis, 23 markers had a 100% call rate and four (APR_DHsnp1854, Lr34Exon22Jag, Wsm2_SNP6660 and Wsm2_SNP80940) had lower call rates because some samples had fewer than 10 read counts.

### Genotype Concordance

GBMAS genotype data were compared to that of KASP or STS markers to validate the accuracy of GBMAS calls. Genotype concordance was calculated as the proportion of matched calls between two marker systems in the total number of DNA samples compared. The concordance between GBMAS and KASP or STS data was 100% for 23 markers including: APR_DHsnp1854, Fhb1_Cg8, Fhb1_319, Lr34Exon11, Lr34Exon12, Lr34Exon22Jag, Lr42_TC425250_08, Lr46_Yr29_JF2, Rht1B, Sr35_CNL9, Sr39_Seg5, Sr39_Sr40_Seg4.1, PHS4A_34562_92, PHS4A_34586_92, PHS4A_8081_92, TaPHS1_3AS, Wsm2_SNP6660, Wsm2_SNP80940, SBM_SNP198467, SBMV_JD_c4438_5568170, Bx7oe, csSr2, and Uhw89.

For four additional markers, most samples were validated by KASP or STS data. For the marker Lr34Intron4, disagreement was only found in one sample. For the STS marker Umn26 for *GluD1*, all samples gave the same genotype calls as GBMAS, except lines AUS591451, AUS591452, RWG1 and RWG4 which did not amplify any PCR product in the STS assay. Marker APR_DHSnp1304 had a relatively low genotype concordance (79%). There were five samples scored as non-APR in GBMAS, but appeared heterozygous in the KASP assays. For marker HF6B-2475, all samples tested positive by GBMAS (monomorphic), but five genotypes were scored negative using KASP assays.

The amplicon for GBMAS marker Sr39_Sr40_Seg4.1 contains three different SNPs that can detect the presence of both stem rust resistance genes *Sr39* (SNP54 and SNP108) and *Sr40* (SNP116) [39]. KASP assays for each individual SNP were designed for use in marker validation. The KASP assays for SNP116 (*Sr40*) and SNP108 (*Sr39*) failed and could not be used to validate the GBMAS data. However, as expected, only the positive controls were scored homozygous positives based on the GBMAS data. The SNP54 site did have good genotype concordance between KASP and GBMAS assays.

## Discussion

Next generation sequencing has been used for SNP identification and genotyping. In this study, we attempted to develop a multiplex assay using next generation sequencing to analyze a set of SNP, indel and CAPS markers commonly used for MAS in hard winter wheat [5,14,19,20,22,26]. Markers for important wheat traits in the Great Plains used in this study include those for wheat resistance to multiple diseases and insects, end-use quality, and plant height. The multiplex assay will allow the simultaneous genotyping of important agronomic traits in bread wheat at a low cost. Although only 32 markers were evaluated in this study, they represent nearly all markers that are currently available to be converted into a GBMAS assay and are associated with traits of interest in U.S. HWW. As more SNPs, CAPS, and indels are discovered and developed, this assay will be quickly expanded.

For a multiplex PCR, optimum primer concentrations may significantly affect PCR amplification efficiency. Primer concentration adjustment is critical to generate enough and relatively uniform amounts of PCR products for sequencing. We started with an equimolar primer concentration of all locus-specific primers (100 nM) and found that a primer concentration of 2-tiers (8 nM and 16 nM) provided relatively uniform read counts and more reads per marker across all markers. The more efficient primers were adjusted to a lower concentration and the less efficient ones to a higher concentration. The overall primer concentrations in our 2-tier multiplex PCR assay are considerably lower than the 50 or 200 nM primer pools used in the commercial Ion Ampliseq Panels (Ion DNA Library Preparation Guide, Life Technologies, Carlsbad, CA).

DNA concentration can be an important factor that causes inconsistent results among samples. Theoretically, normalized DNA should provide a more uniform PCR amplification across different samples. In this study, we found an extremely high correlation (R^2^ > 0.92) in read counts between normalized and non-normalized DNA, suggesting that DNA normalization may not be necessary within the DNA concentration range used (17 to 141 ng/reaction). The use of non-normalized DNA would make GBMAS library construction easier, faster and cheaper as long as proper quality and quantity control is made during the sample collection and DNA isolation process. However, a much wider range of DNA concentrations across samples will likely result in a high number of reads for some samples with high DNA concentrations and a low number of reads for those with low DNA concentrations, which could lead to incorrect genotyping interpretations, especially when heterozygotes need to be distinguished from homozygotes. Also, more missing data will be present in samples with an extremely low DNA concentration. The elimination of a DNA normalization step for GBMAS assays is possible if care is taken to use equal amounts of tissue across samples. Automated DNA extraction systems for DNA isolation can further reduce variation in DNA concentrations among samples.

TD PCR improves PCR amplification specificity and sensitivity [40] and has been reported to ameliorate multiplexed microsatellite and SNP genotyping assays [41,42]. However, in this study, the non-TD protocol gave a more consistent read count among PCR and sequencing runs than the TD protocol. This is in agreement with the multiplex PCR assay in the Ion Ampliseq Cancer Panels (LifeTechnologies, Carlsbad, CA) and the Sequenom MassArray (Sequenom, San Diego, CA) protocols which both also use non-TD PCR. TD PCR may be more suitable for uniplex than multiplex PCR because multiplex PCRs have many different primers that often differ in optimum annealing temperature, even if they were designed for the same target melting temperature. Primers with relatively higher annealing temperatures will amplify at earlier cycles in TD PCR and negatively affect the yield of, or simply amplify more efficiently than, those with a lower optimum annealing temperature.

The minimum read counts per sample-marker combination was set to 10 reads to minimize the effects of sampling bias on genotype calls. This minimum criterion is similar to the cutoff used by Rife et al. [36]. However, when the cutoff was lowered to 5, many false positive calls appeared. Dominant markers with null genotypes, the indel marker for *Bx7oe* and the alien fragment-derived markers for stem rust resistance genes *Sr2* and *Sr35* for example, are exceptions to this cutoff because a zero read count for these markers implies that the sample carries the deletion or null allele. However, a true null allele or deletion is not distinguishable from false negatives caused by PCR reaction failure. Thus, null allele data should be interpreted with caution.

We analyzed 24 different putatively homozygous genotypes with four single plant replicates each. We found consistent data across all four replicates for 23 genotypes. WGRC27 had different genotype calls for some markers. This is most likely due to heterogeneity from the breeding or seed increase processes. Because the selected genotypes used are all cultivars or released germplasm, they were all assumed to be homozygous. Thus, the plants were scored as either homozygous positive or homozygous negative based on the minimum percentage of favorable allele reads. The minimum read percentage of a favorable allele (cutoff) varied with markers and across runs in general. Therefore a universal cutoff is not available. Control samples must be included in each GBMAS run and the sequence read percentages of the favorable allele in the controls should be used to set the cutoffs for homozygous positives. If heterozygous genotypes need to be determined, a synthetic heterozygote control with an equal mix of homozygous positive and negative control DNAs may help set the cutoff values for differentiating heterozygotes from homozygotes; however, genotype calls would probably still be complicated due to the polyploid nature of wheat [16,32]. The minimum sequence read percentages of favorable homozygous alleles was 50% or lower for most markers in this study, which agrees with the theoretical percentage of expected favorable alleles resulting from the amplification of alleles from two or three genomes (Table 1). For heterozygotes, the minimum percentage is even lower in wheat and varies with the genome specificity of primers (Table 1), thus, even with large numbers of sequencing reads, the differences in cutoff value between a heterozygous favorable allele and its homozygote can be too small to be clearly separated in single plants from early generation breeding populations. This limits the certainty of homozygous positive genotype calls when using GBMAS in wheat to homozygous materials such as varieties, advanced breeding lines and double haploids. The GBMAS method is useful for heterozygous and heterogeneous materials to determine if certain alleles are present, though the exact zygosity level may be impossible to determine.

In hexaploid wheat, designing genome-specific primers can help separate a heterozygote from its homozygote. PolyMarker, a polyploid primer design program [43], can be used to align target sequences to the survey sequence of wheat var. Chinese Spring [44] to identify genome-specific variants (if available) prior to designing KASP primers. Ramirez-Gonzales et al. [45] used this program to design primers for 35 wheat yellow rust (*Yr15*) SNPs and obtained 17 genome-specific, seven semi-specific and nine non-specific KASP primers. This result underscores the difficulty in designing genome-specific primers in hexaploid wheat even with the recently released wheat reference genome [44] because of the high sequence similarity between genes in the three wheat genomes and the lack of available homoeologous sequence information from different varieties. GBMAS primers were designed tens of bases away from the site of interest. This is an advantage over other genotyping platforms because GBMAS primers can be located at the site of interest or up to hundreds of bases away. SNaPshot and Sequenom are single-base extension assays and depend on the availability of a suitable extension primer with a 3’-end that anneals one base directly upstream of the base to be detected. Likewise, the 3’-end of the allele-specific KASP primers have the same restriction on primer design. Given the primer position requirement of these assays and the presence of only two DNA strands, primers for these genotyping assays can only be designed at two possible positions and often no suitable primers can be found. In contrast, the presence of sequence variants neighboring the site of interest is not a hindrance in GBMAS. Since GBMAS primers are typically designed away from the site of interest, an amplicon can contain more than one unique site, as exemplified by the Fhb1_Cg8, Sr35_CNL9, TaPHS1_3AS and Sr39_Sr40_Seg4.1 markers. The first three markers contain two SNPs each. The Sr39_Sr40_Seg4.1 amplicon is unique in that it has two SNPs unique to resistance gene *Sr40* and one SNP for resistance gene *Sr39*.

Among 27 GBMAS markers that were successfully amplified, 23 were in complete agreement with their corresponding KASP or STS assays. This percentage (85%) is higher than that reported by Berard et al. [32] who ran 11 SNPlex markers on 42 lines and validated seven as having a perfect match using Sanger sequencing and fully verified only three markers using the MassArray genotyping platform. In this study, concordance between other assay types and GBMAS was excellent; however, non-concordance was found in some genotypes for four markers. For instance, discrepancies were found in five genotypes for HF6B between GBMAS and KASP assays, and in four genotypes for the *GluD1* marker using GBMAS and STS assays. The disagreement in genotype calls for HF6B might be due to non-genome-specific amplification and highly similar sequences among the three wheat genomes that masked the true allelic ratios and genotypes, whereas the disagreement in the *GluD1* marker could be due to a polymorphism or indel in the sequences that prevents proper primer binding in these samples, and redesigning primers that bind to a different location of the marker DNA sequence may solve this problem.

We have developed a multiplex assay for 27 markers suitable for MAS in wheat. The GBMAS assay had excellent concordance with both KASP and STS assay data and should be as reliable as those two corresponding uniplex methods. This core set of markers is suitable for MAS of advanced breeding materials, double haploid lines, inbred parent lines, and released cultivars. This assay is also useful in heterozygous and heterogeneous materials if exact zygosity levels are of secondary importance. SNP and other marker discovery in wheat is an on-going and continuing effort. As new and better markers are discovered, additional primers will be designed and added to this core set. Moreover, bioinformatic analysis of GBMAS sequence data, coupled with alignment to all three Chinese Spring reference subgenomes, will identify new sequence variants and generate more discriminating and genome-specific primers for single genes as well as QTL-associated marker haplotypes for use in GBMAS. Finally, GBMAS is less expensive, more flexible, more rapid, more reliable, and more informative than using individual SNP markers or most chip-based assays.

## Supporting Information

S1 Table Primer sequence of GBMAS markers. (DOCX)
