# Supplementary material for: Using Next Generation Sequencing for Multiplexed Trait-Linked Markers in Wheat
Source: PLoS One. 2015 Dec 1;10(12):e0143890. doi: 10.1371/journal.pone.0143890 (PMC4666610; doi:10.1371/journal.pone.0143890)
Supplement: S1 Table — (DOCX) [file pone.0143890.s001.docx]

| **Table S1. Primer sequence of GBMAS markers** | |  |  |
| --- | --- | --- | --- |
| **Marker name** | **Forward primer sequence*** | **Reverse primer sequence**** | **Amplicon length** |
| BX7oe | TCACGCCATCACTAATATTC | TAGTTTTAGTTCTCATATCC | 136 |
| csSr2 | CCATTGCTCTACACAACAAG | CTTTAGCAGCGTTACTTCC | 149 |
| APR_DHsnp1304 | CATTGCCATGAACATCCTG | ACATTTTCTCCCACTCCAC | 150 |
| APR_DHsnp1854 | CCAGGAGCAATGCCAATAC | CCGTTCCTGGCAATGGTG | 93 |
| Fhb1_319 | ACCAGTTGTACTGAATTCAG | GTGTTACGAGGTATTGACC | 133 |
| GluD1_umn26_4777 | GTACTAGCACGATAGTCAC | TATATGCATCCGTGTCTCG | 150 |
| HF1A-5150 | CCACAAAAGATGGCTTCTG | CAGTTTAAAGAAGCTCTCGC | 147 |
| HF6B-2475 | ATTGTGTGTGTTGAGCTCG | TCGGTGTGATTCAAATGGG | 161 |
| Lr21_InDel_R | CCCTTGTTTATTATTTACAC | TGTCTGTCACATGTCTATTC | 168 |
| Lr34Exon11 | ATCTGCATGCTCATCTCTG | GCTTGCCATTATTGCACTC | 150 |
| Lr34Exon12 | GTTCTTCTGCCAGTTTCTG | TGAAGCCTCCGAATGTAAG | 149 |
| Lr34Exon22Jag | TGAAAGCCTGTAGAAGGTG | GCGTATTGTAATGTATCGTG | 153 |
| Lr34Intron4 | TAACCATGACACCGACATC | GTGTTGAATCTCAGGATGC | 148 |
| Lr42_113325_01 | TCATTGGGTGACCTAAGAC | CTCCTTCCTCATATGAGAC | 151 |
| Lr42_TC425250_08 | TCTGAGTCTTCACAAACGC | ACCTAGGTATTCAGAGTGG | 150 |
| Lr46_Yr29_JF2 | CCATCTTAATATGCAACTCG | CTTGGAGCGTTGGTATTAAG | 148 |
| PHS4A_34562_92 | TCCATGCATCATAGGAAAAC | GCTTCTGTCCCTTACTTTG | 150 |
| PHS4A_34586_92 | TTCTCGTGCTTGTCGGTC | TCGTTGATCGCGCACAATG | 107 |
| PHS4A_3743_9 | CATTGGGCTTTGCATCTTG | CAACAGGTATTGTACCGAC | 150 |
| PHS4A_8081_92 | TGGTGTTGCAAAAACAATGG | AATCAGAACCCATCGCCC | 101 |
| Rht1B | TGGGTGGCGAAGCTGTC | TCGGGTACAAGGTGCGG | 124 |
| SBM_SNP198467 | TCATCGAAACAACGAGAGG | CACTGCAATGAGTAGGTAG | 149 |
| SBMV_JD_c4438_5568170 | TGCCTTCACAAGTTCTCTG | TTTTTGTCGTTGAGGTTTCC | 152 |
| Sr35_CNL9 | TAGCTCCCTCACTGATAAC | CTATGTTTCCACAAGGAGC | 150 |
| Sr39_Sr40_Seg4.1 | AACAGTGCTTCTTAGAAAGG | TGATAAAGCCACATGACGG | 153 |
| Sr39 _Seg5 | ACAGGAAAAAGAGGCAGAG | GCTAGCTAGCATGTGAATG | 190 |
| TaPHS1_3AS | GTGCAATTTACATGTGAATGG | TTGTGTCTGCAGGATAGTG | 146 |
| Uhw89 | TTATGGGAGTAGGTTGGTG | CTAAGAAAGGGAGAGAGAG | 149 |
| Umn10 | TTCATTGCCACGCATATGC | TGGTTCCACGTCTTCTTAC | 233 |
| Wsm2-SNP6660 | ATCACAGCCCTGAAGGAC | TCGGTGTTCAGCTTTCTTG | 88 |
| Wsm2-SNP80940 | TGGAGGTTATTTGACGGAG | TGGACATTCTGTCTCTTCG | 151 |
| Yr17-Lr37-Sr38 | CTTGCAGATGAGGAGGAG | CTCTCAGCTTCACAGACTG | 102 |
|  |  |  |  |
| *add M13 tail GATGTAAAACGACGGCCAGTG to 5'-end of forward primer | | |  |
| **add CCTCTCTATGGGCAGTCGGTGAT to 5'-end of reverse primer | | |  |
